# Development of a spatially targeted field sampling technique for the southern cattle tick, Rhipicephalus microplus, by mapping whitetailed deer, Odocoileus virginianus, habitat in South Texas

**DOI:** 10.1093/jis/14.1.88

**Published:** 2014-07-08

**Authors:** Pamela L. Phillips, John B. Welch, Matthew Kramer

**Affiliations:** 1 USDA-ARS, SRU, KBUSLIRL, 2700 Fredericksburg Rd., Kerrville, TX 78028; 2 USDA/APHIS IS Screwworm Eradication Program, Pacora, Rep. of Panama; 3 USDA/ARS BARC, BSC, 10300 Baltimore Ave. Beltsville, MD 20705

**Keywords:** GIS, satellite remote sensing, habitat mapping

## Abstract

The objective of our study was to determine whether satellite remote sensed data could be used to identify white-tailed deer, *Odocoileus virginianus* (Zimmerman) (Artiodactyla: Cervidae), habitat and target locations for sampling free-living larvae of the southern cattle tick, *Rhipicephalus (Boophilus) microplus* (Canestrini) (Ixodida: Ixodidae) in South Texas. Two methods for mapping white-tailed deer habitat were used, an object-oriented method to identify closed canopies and waterways for deer movement and two vegetation indices: the Normalized Difference Vegetation Index and the Modified Soil Adjusted Vegetation Index to identify forage for deer. These two data sets of favorable white-tailed deer habitat were combined within a geographic information system to identify locations for sampling ticks. Larvae of *R. (B.) microplus* , were sampled in Zapata County, Texas, by walking transects with attached flannel panels to jeans. Although the data set and sampling period were limited, data analysis demonstrated that sampling of free-living larvae of *R. (B.) microplus* can be conducted in South Texas, and larvae were most abundant in areas that harbored *O. virginianus* . Spatial analysis of satellite imagery to classify white-tailed deer/southern cattle tick habitat proved efficacious and may be useful in directing sampling activities in the field.

## Introduction

Southern cattle tick, *Rhipicephalus (Boophilus) microplus* (Canestrini) (Ixodida: Ixodidae), were eradicated from the Southern United States predominantly by systematically treating all bovines and equines with a topical acaricide every two weeks until all animals were free of ticks, or by removing all bovines and equines from infested pastures for a period long enough for tick larvae to die from lack of hosts ( Mohler 1942 ). Pound et al. (2010) presented substantial evidence that increasing incidences of premise infestation both within and adjacent to the Systematic Area of the Cattle Fever Tick Eradication Program (CFTEP) in South Texas were attributable to infestations on white-tailed deer, *Odocoileus virginianus* (Zimmerman) (Artiodactyla: Cervidae). Knowledge of the ecology and biology of the target species is critical for the design and implementation of tick-eradication strategies ( George et al. 2002 ). Our study reports the use of satellite remote sensing and field sampling techniques for collecting immature southern cattle ticks and modeling their habitat use in South Texas in the context of white-tailed deer habitat use.

*Rhipicephalus microplus* is a one-host tick; once the larvae attach to a host, they mature from nymph to adult on a single host. The off-host and free-living phases of this tick’s life cycle are the preoviposition, oviposition, incubation, eclosion, and free-larval stages ( Legg 1930 ; Hitchcock 1955 ; Nuňez et al. 1982 ). Of these stages, the host seeking larval stage is more easily sampled, as the other stages are generally concealed within the vegetation ( Wilkinson 1961 ). *R. microplus* larvae are small, approximately 500 μm long and 400 μm wide, and not easily located by visual examination ( Nuňez et al. 1982 ). After eclosion, the larvae tend to aggregate and climbonto vegetation, preferring the shaded side, and ascend to heights between 2.5 and 85.1 cm ( Garris and Popham 1990 ). Wilkinson (1953) reported this height to be specific for the principal host, domestic cattle.

Although the southern cattle tick is an introduced species, Pound et al. (2010) demonstrated the historical role of white-tailed deer for the last 35 years in South Texas and how they contribute to the maintenance of cattle fever tick populations and to the dispersal of ticks, requiring changes in livestock management practices. *Odocoileus virginianus* are a recognized host of *R. microplus* and *R. annulatus* and are able to distribute engorged female ticks within their home ranges, which can be approximately 264 ha for bucks and 189 ha for does ( Pound et al. 2010 ). This greatly increases the extent and scale of exposure and potential infestation by cattle fever ticks, which has traditionally been limited to premises immediately surrounding an infested property (i.e., *O. virginianus* routinely cross three-strand barbed wire fences that generally confine cattle within a premise). *Odocoileus virginianus* have been proven to be capable of maintaining a reservoir population of ticks, thus impeding the success of the cattle tick eradication program in South Texas ( George 1990 ; Pound et al. 2010 ).

Predictive and simulation models have been developed for the southern cattle tick, *R microplus,* for large geographical areas that encompass entire continents (Estrada-Pena et al. 2005, 2006) as well as for smaller geographical areas using microclimates and habitats of South Texas ( Teel et al. 1996 ; Corsan et al. 2004 ). Estrada-Pena et al. (2005) developed spatial environmental niche models using almost fifty years of climate data to identify changes in habitat suitability over time for *R microplus* in the Americas and to forecast future southern cattle tick habitat suitability for these continents. For the USA and Mexico, the authors found a clear association of precipitation and humidity derived variables with tick habitat suitability. Estrada-Pena et al. (2006) further developed the model for Mexico and incorporated the Normalized Difference Vegetation Index (NDVI) from satellite remote sensing data. In both models the habitat suitability for ticks was based on tick infestations and not free-living ticks. Teel et al. (1996) developed simulation models incorporating cow-tick-landscape interactions. Corsan et al. (2004) strengthened the model by adding the climatic factors for each of the habitat types in South Texas. Both of these studies used cattle as the sole host and their use of the landscape to develop the models. None of these studies included the impact of *O. virginianus* as hosts of cattle fever ticks and their effect on their distribution.

Long term surveys of off-host ticks have proven beneficial in understanding seasonal activities of ticks and lead to the development of time-series analysis of environmental factors that influence tick abundance and prediction data ( Spickett 1994 ). Sampling for off-host larvae of *R. (B.)* spp. in South Texas was previously considered unreliable and impractical due to the topography and the dense brush habitat ( Teel et al. 2003 ). However, studies in Australia and Mexico have shown that cattle ticks can be sampled off the host animal ( Blakeslee and Bruce 1948 ; Wilkinson 1953 , 1957, 1961; Wilkinson and Wilson 1959 ; Fernandez et al. 2003 , 2004). Off-host sampling of other tick species in brush and forested habitats have been associated with animal trails ( Schulze et al. 2001 ).

In our study, favorable deer habitats were identified and incorporated to sample for *R. microplus* and, when possible, deer trails were followed within the habitat types. Fulbright and Ortega (2006) described the optimum deer habitat to be a patchwork of varying vegetation types that provide cover, forage, and access to water. The spatial relationship of these data were identified using satellite remote sensing and integrated into a geographic information system to identify favorable *O. virginianus* habitat. In South Texas, rivers and streams provide the major source of water, as well as a dense vegetative cover. Tree covered waterways provide shelter, and adjacent mixed brush vegetation provides suitable deer forage. In the study area, riparian vegetation was mapped and identification was made of nearby or adjoining brush, forbs, and grasses described and identified by Fulbright and Ortega (2006) as potential available forage.

Areas favorable to *R. microplus* larval tick field survival are determined by temperature and humidity requirements ( Davey et al. 1991 ). The type of vegetation affects the microenvironment, making a site favorable or unfavorable for larval survival ( Fleetwood and Teel 1983 ; Sutherst 1983 ; Teel 1984 ; Garris and Popham 1990 ). Teel et al. (1997) and Davey et al. (1994) characterized South Texas habitats of canopied mesquite and mixed-brush vegetation as more favorable for higher numbers and longer survival of cattle fever ticks than grass and grass-mixed brush. The dynamics of tick infestation and occurrence is a complex function of many factors, which may include the degree of contact between cattle and white-tailed deer, climatic variables, and geographic locations to habitat and water. Geographic information systems and satellite remote sensing can map interactions between vectors, hosts and habitat, and spatially target high-density foci for deer and tick larvae.

One objective of this study was to determine whether a field method of sampling *R. microplus* larvae could be developed for the South Texas ecosystem using habitats favorable to *O. virginianus* to target field sampling sites for free-living tick larvae. Satellite remote sensing images were used to identify probable areas where populations of *O. virginianus* could be found and collect field data to describe these habitats. A second objective was to determine whether the sampled tick data followed a pattern that could be predicted from habitat variables and satellite remote sensing data. The data sets for these analyses are presented in Table 1 and described in more detail in the following sections.

**Table 1.**
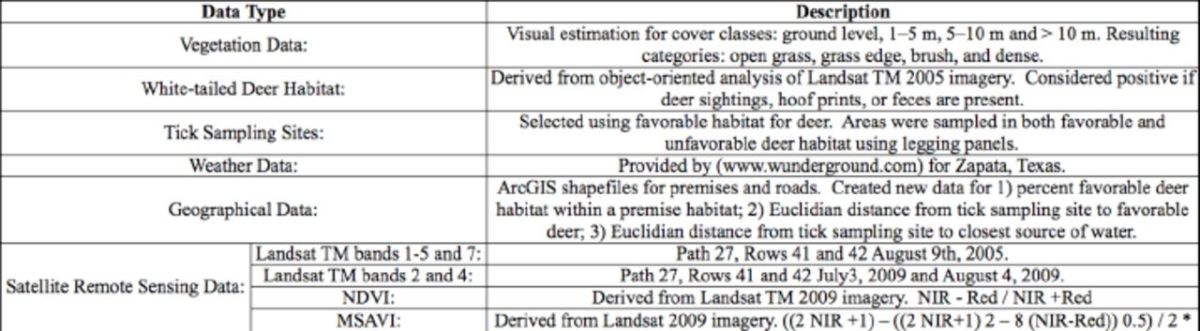
Description of data types used in the study.

Red = Band 3, NIR = Band 4 on Landsat 5 TM imagery

## Materials and Methods

### Study area

**Figure 1.**
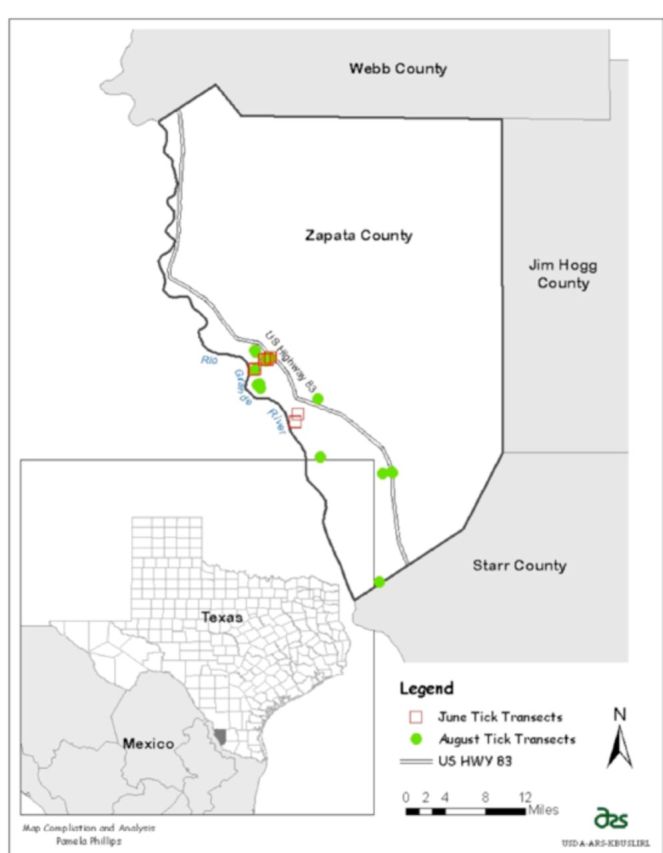
Location map of study area and tick sampling locations for June and August 2009. High quality figures are available online.

Zapata County is located in the South Texas Plains ecological area ( Gould et al. 1960 ), partially within the CFTEP permanent quarantine zone. The quarantine zone is defined by those areas south of Texas Highway 83 ( Figure 1 ). An area of approximately 265 square kilometers within 12 premises was sampled, with one premise located north of Highway 83. The climate is subtropical steppe with semiarid to arid conditions. The average rainfall is approximately 50.8 cm per year with seasonal peaks in May, June, and September ( Buckley and Dodd 1969 ). Thornthwaite (1948) classified the vegetation in South Texas as subtropical semiarid vegetation. Along the Rio Grande River, the flora is typically subtropical woodlands with a low growing canopy consisting of mesquite, *Prosopis glandulosa* (Torrey); granjeno, *Celtis pallida,* (Torrey); huisache, *Acacia fernesiana* (L. Wild); catclaw, *Acacia greggii* (A. Gray); shrubby blue sage, *Salvia batllotiflora* (Bentham); blackbrush acacia, *Acacia rigidula* (Bentham); cenizo, *Leucophyllum frutescens* (Berlandier) I.M. Johnston; guaycan, *Porlieria angustifolia* (Englemann) Gray; and prickly pear cactus, *Opuntia lindheimeri* (Englemann) interspersed. Another component to the vegetation in South Texas is an introduced species of bufflegrass, *Cenchrus ciliaris* L *.* , which is an aggressive perennial grass invading native habitats. McMahan et al. (1984) mapped the vegetation types in Zapata County as a combination of Ceniza-Blackbrush-Creosote bush-Brush, Mesquite-Blackbrush Brush, and Mes-quite-Granjeno assemblages.

Zapata County was selected for this study because of the historically high cattle tick infestation rates and the abundance of *O. virginianus* . The premises selected for sampling were determined in part by the ability to obtain consent from land owners to access and sample on their property. Once ticks are collected within a premise, that area is designated “infested” by the regulating agency, USDA-APHIS, Veterinary Services (VS), CFTEP. Once a premise is classified as infested, the cattle on the premise are then subjected to treatments by the CFTEP ( George et al. 2002 ). *Rhipicephalus microplus* in Zapata County have been a serious problem for land owners, which afforded cooperation, consent, and access to the sampled properties by the owners.

### Landsat 5 image acquisition and processing

Prior to collecting field data, two Landsat 5 Thematic Mapper (TM) ( http://landsat.usgs.gov/ ) images processed with the Standard Terrain Correction (L1T) (path 27, rows 41 and 42) were acquired of Zapata County for 9 August 2005 for direct download from the USGS Earth Observation and Science enter (EROS) website (glovis.usgs.gov). The L1T data have an ap proximate scene size of 170 km north-south by 183 km east-west and consist of seven spectral bands with a spatial resolution of 30 m. For this study, TM bands 1 (0.45–0.52 µm), 2 (0.52–0.60 µm), 3 (0.63–0.69 µm), 4 (0.76–0.90 µm), 5 (1.55–1.75 µm), and 7 (2.08–2.35 µm) were used to determine favorable deer habitat. The geographic projection used for all spatial data sets was the Universal Transverse Mercator (UTM) Zone 14N datum WGS-84 projection. The Landsat 5 TM sensor was in operation from July 1982 to May 2012 with a 16-day repeat cycle. The 2005 satelliteimagery was used to classify the riparian vegetation and to select areas to field sample for deer presence in 2008. Four additional Landsat 5 TM images with the same path and row for 3 July 2009 and 4 August 2009 were acquired from the EROS website. These were the two dates closest to the time of field sampling for ticks and with the least cloud cover.Radiometric correction was performed on the 2009 images using the Erdas Imagine ^®^ 9.3add-on module ATCOR2 ( www.hexagongeospatial.com ). ATCOR2 uses sensor specific look-up tables from MODTRAN-4 code to correct for solar illumination and sensor viewing geometry to obtain true surface reflectance ( Richter 2008 ). Atmospheric correction is an important part of pre-processing when comparing and analyzing multi-temporal images. An image mosaic and subset were also created using the methods described above.

The extent of the study site was at the intersection of two Landsat paths, so an image mosaic was performed of the two scenes and a subset created for Zapata County, Texas, using Erdas Imagine ^®^ 9.3. Favorable deer habitat was mapped using three different methodologies. The Erdas extension VLS Feature Analyst ^TM^ (FA) (Visual Learning Systems 2004) was used to extract riparian vegetation and waterways that could serve as cover and paths for deer population movement, and two vegetation indices (VI) were calculated with Erdas Imagine ^®^ 9.3 to map other vegetation types important to deer, in particular suitable brush vegetation for deer forage. VI are used in satellite remote sensing to provide quantitative and qualitative analysis of vegetation cover by contrasting intense chlorophyll pigment absorption in the red wavelength to the high reflectance of vegetation in the near infrared wavelength ( Tucker 1979 ).

VLS Feature Analyst ^TM^ uses an object-oriented classification method to partition satellite remote sensing data into a given object class by using characteristics of texture, shape, relative size, and groups of pixels in a context that are spatially related ( Bruce 2008 ). Visual interpretation of riparian vegetation and rivers were digitized and used to train the FA algorithm to identify these throughout the 2005 TM imagery. NDVI and MSAVI are two commonly used ratio based global VI. The upper bound for these ratio indices are 1 with the lower bound of -1. Negative values correspond to water, values close to zero to barren rock or sand, low positive values to shrubs and grasslands, and high values to dense vegetative cover. For our study, both indices were derived from the Landsat 5 TM 2009 imagery using bands 3 (red wavelength) and bands 4 (near-infrared wavelength). A combination of riparian vegetation mapped using Feature Analyst along with VI values for brush and wooded grasses were identified as forage and considered favorable for white-tailed deer. Many studies have shown these indices reflect the abundance and vigor of green vegetation ( Rouse et al. 1973 ; Richardson and Wiegard 1977 ; Tucker 1979 ; Clevers 1989 ). VI have been related to leaf area index, degree of plant cover, chlorophyll content, green biomass, and even absorbed photosynthesis effective radiation ( Baret and Guyot 1991 ; Jackson and Huerte 1991 ). Hess et al. (1996) found NDVI useful for discriminating semiarid vegetation; however, it does not account for the soil background (Huerte 1988, 1989 ). Soil background affects the overall vegetation reflectance and is more problematic in semiarid environments, such as in the brushland of South Texas. The MSAVI equation was used to calculate for a soil adjustment factor by using an iterative factor to minimize the soil effect ( Qi et al. 1994 ; Chehbouni et al. 1994 ; Jiang et al. 2007 ). The equation for each index is:

NDVI = NIR - Red / NIR + Red

MSAVI = ((2 NIR +1) – ((2 NIR+1) ^2^ – 8 (NIR-Red)) ^0.5^ ) / 2

Extraction of VI values for vegetation field sites and tick sampling sites were done using ESRI ArcGIS ^®^ 10 ( www.esri.com ).

### Geographical data

Use was made of ArcGIS ^®^ 10 digitized shape-files of the property boundaries developed by the USDA ARS Knipling-Bushland US Livestock Insects Laboratory (Kerrville, Texas) in collaboration with CFTEP to determine the number of hectares and to calculate the percent of favorable deer habitat within a premise where tick larvae were sampled. The favorable deer habitat was mapped using three different satellite remote sensing methodologies described previously: object oriented classification, NDVI, and MSAVI. Calculations were also made of the Euclidian distance of tick sample sites to the nearest favorable deer habitat and to water sources using the Spatial Analyst Extension of ArcGIS ^®^ 10. These data sets were incorporated in the statistical modeling.

### Field sampling for deer habitat

Classification of habitats hypothesized to be favorable for deer was conducted as described above. Ground verification of deer habitat and collection of GPS coordinates, using a hand held Garmin eTrex Vista CX ( www.garmin.com ), was conducted on 6 and 7 May 2008. Habitats were classified as positive for deer if deer were sighted, hoof prints present, or deer feces were present. To determine which vegetation indices performed best in distinguishing between the habitat types, 100 GPS points were collected of the varying vegetation cover in Zapata County along State Highway 83 using the Garmin GPS unit (June 23–26, 2009) and later using a Delorme Earthmate PN-40 ( www.delorme.com ) (August 10–12, 2009). The locations were selected randomly for large contiguous areas of vegetation types differing in species composition and varying in canopy coverage. The approximate percentage of vegetation cover and dominant species composition were described for each of these locations by making a visual estimation for the cover classes by height for: ground level, 1–5 m, 5–10 m, and greater than 10 m.

### Field sampling for tick larvae

**Figure 2.**
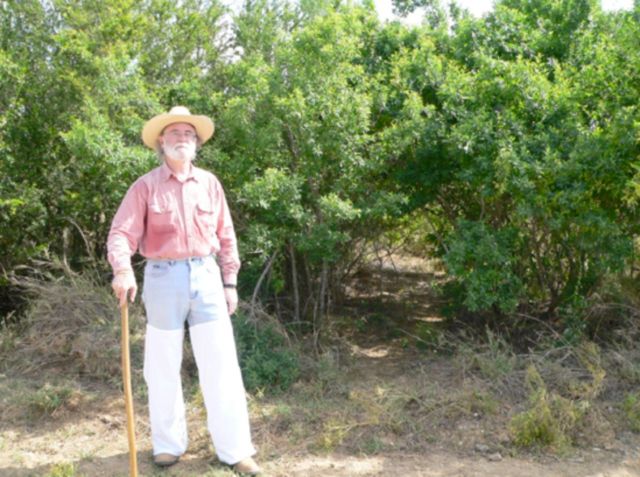
Photo of tick sampling leggings and example of favorable habitat and deer trail (site June 5). High quality figures are available online.

**Figure 3.**
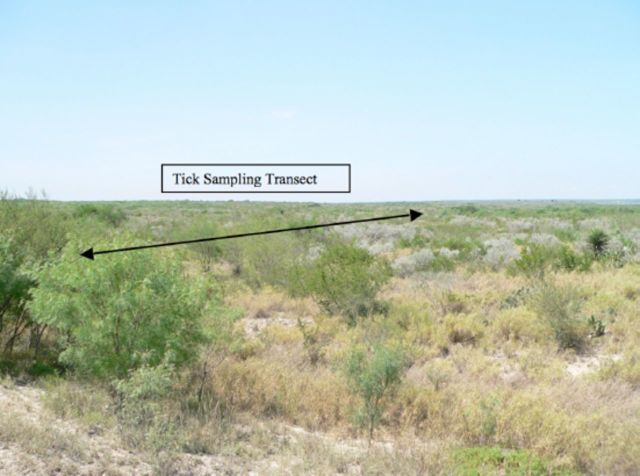
Tick sampling site with the highest number of larval *R. microplus* . The line indicates the approximate location of the transect. The site was heavy, thick brush habitat (site June 10). High quality figures are available online.

Larval tick sampling was conducted on 23–26 June and 10–12 August 2009. Table 2 provides the list of sample locations, the vegetation type, and the number of tick larvae. Eleven sites were sampled in June (June1–11) and 21 sites were sampled in August (August1–21). A white flannel (Super Flannel 0030, Wal-Mart, www.walmart.com ) panel, 67.3 cm wide x 74.9 cm tall, was attached by Velcro to each leg of a pair of blue denim pants ( Figure 2 ). Since walking and flagging samples can be biased by differences between investigators ( Ginsberg and Ewing 1989 ), the same person sampled for ticks at all sites. Sampling was initially conducted by walking approximately 7.5 minutes through the selected habitat. This was done on 23 June. As less ground was covered when travelling through dense vegetation compared with open pasture, the procedure was then modified by incorporating GPS positions to extend sampling through the habitats with dense vegetation ( Figure 3 ). By incorporating this method, we were able to maintain a similar length of approximately 200 m for all habitats.

**Table 2.**
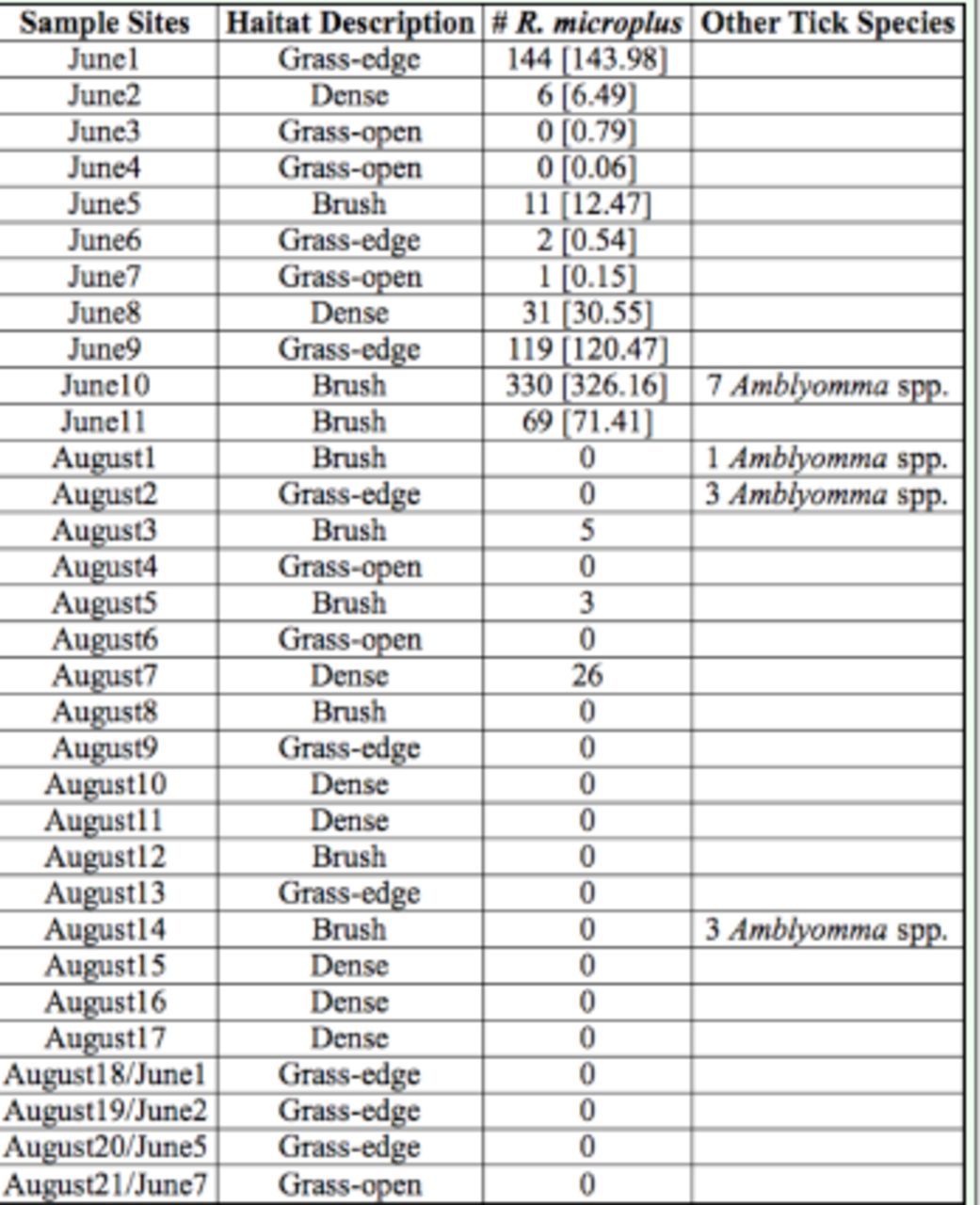
Sampling locations in Zapata County, Texas for June and August 2009 for *R. (Boophilus) microplus* larvae.

Numbers in square brackets for June samples are fitted tick counts from a generalized linear model with the following independent variables: habitat type, Modified Soil Adjusted Vegetation Index (MSAVI), distance from vegetation cover, and a dummy variable indicating whether the samples came from Volpe Pastures (June 9, 10, 11). Note that the June 1 count sums over two samples.

Sampling was conducted by walking outbound from and returning to a predetermined starting point for a combined distance of 400 m. Immediately upon return to the starting point, the panels were removed from the pants and examined for the presence of tick larvae. Each panel was examined once by both field investigators and then an additional time to verify all tick larvae were detected and removed. Tick larvae were placed in labeled vials containing 100% ethanol and transported to the USDA-APHIS-Veterinary Services, CFTEP, office in Zapata, Texas. The samples were turned over to program personnel for preliminary identification and subsequent shipment to USD A-APHIS, National Veterinary Services Laboratory in Ames, Iowa, for final identification and verification according to CFTEP protocol.

The sampling panels used on 23-26 June were carefully examined and then reused. Field sampling during 10-12 August used one set of panels per collection site. These panels were examined, and ticks were collected and preserved as detailed above, but then placed in locking plastic bags, labeled per site, and transported to the CFTEP office in Zapata. All panels, in June and August, were sprayed with coumaphos by CFTEP personnel before they were removed from the cattle tick quarantine area.

### Weather data

Temperature, humidity, dew point, and rainfall data for the analysis were downloaded from the Zapata (KAPY) weather station from the website Weather Underground ( www.wunderground.com ). The KAPY is listed with the National Weather Service as an observed weather station. No microhabitat weather data were available for our study. These data were then used as independent variables in the statistical model.

### Statistical model

Tick (and most other arthropod) counts in the field are often modeled as samples from a Poisson distribution, which is the theoretical distribution if animals are distributed at random over the landscape, modified by allowing for over-dispersion (clumping) and by including independent variables that predict how the mean count should change as one moves across locations in the study site. This kind of model is in the generalized linear model (glm) framework, where observations can come from a variety of distributions (including the normal distribution). It was assumed the data were samples from an over-dispersed Poisson distribution, where there is an estimated scale parameter that accounts for the increased variance due to clumping. The link function for this distribution is the log, i.e., the model fits the expected value of the natural log count at each site. Since only 12 sites were available for analysis, independent variables were limited in the model to avoid over-parameterization. In addition, there were very few tick larvae found in August; only June counts were modeled. The GLM function, with family = quasipoisson, in R (R Development Core Team 2011) was used to fit models.

To reduce the number of candidate independent variables, principal components decomposition was performed on the weather variables, as well as for other meaningful groupings of independent variables, and these derived variables were used as candidate predictors. It was determined that these derived variables did not produce a better fitting model (the derived weather variables were not predictive at all for these data); our final model contained only a few independent variables on their original scale and an over-dispersion parameter.

Given the small size of the data set, we restricted ourselves to simple models to avoid over-parameterization and checked that each final variable was an important predictor in all model variants examined, judged by small *P-* values. Akaike information criterion is not available for quasi-Poisson distributions.

## Results

### Vegetation indices

**Figure 4.**
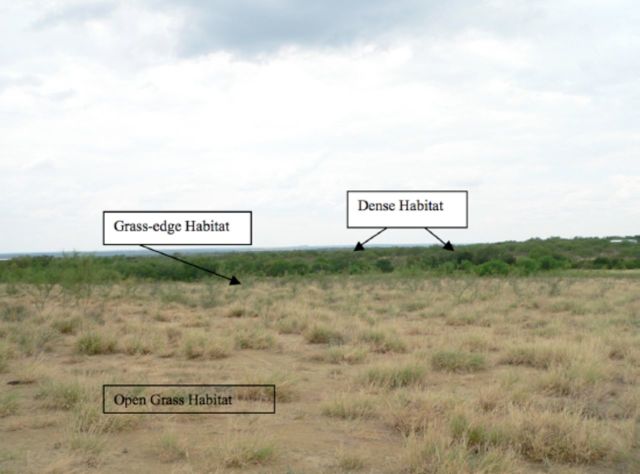
Photo with example of open grass habitat. Note the clumps of grass and the open soil. The edge between grasses and dense usually grades into an area with denser tall grasses grading into the dense vegetation, often a mixture of mesquite and huisache. High quality figures are available online.

Descriptions of the vegetation were visually estimated in the field for percent vegetation cover and categorized into dense, brush, grassedge, and open grass ( Figure 4 ). The grassedge category is based on the association of grass habitat with adjacent brush or dense habitat. The dense category was predominately tree covered vegetation composed primarily of mesquite. Table 3 indicates the range of MSAVI and NDVI values for each category. Previous studies have shown there was a close correlation between vegetation index and the biophysical variables of vegetation, such as fractional vegetation cover, biomass, and leaf area index ( Tucker 1979 ; Huete 1988 ; Clevers 1989 ; Baret and Guyot 1991 ). The overlap in the VI values for the grassedge, brush, and dense categories is probably due to the variation in fractional vegetation cover. Some of the brush vegetation type also had very dense and compact vegetation and expressed higher VI values.

**Table 3.**
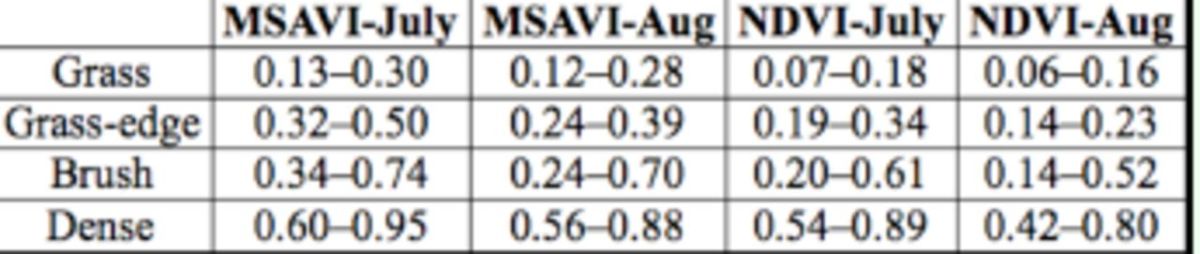
MSAVI and NDVI values for vegetation categories.

Regression models of MSAVI and NDVI with the 100 vegetation sites for both June and August images were compared. Both indices were significantly correlated ( *P* = 0.001) with the vegetation cover and vegetation type observed in the field ( Table 2 ). In linear models, the best fit in July was obtained by using MSAVI with an R2 value of 0.7244. For August, the best fit was also the MSAVI index with an R2 value of 0.6157. NDVI index values for June had an R2 value of 0.6875. August NDVI values had an R2 value of 0.5801. Both indices performed well with MSAVI giving a slightly better fit for both dates. The August VI values are probably less correlated because of the dry grass vegetation, which is highly reflective in both the visible and mid-infrared regions of the spectrum.

### Deer habitat sampling

**Figure 5.**
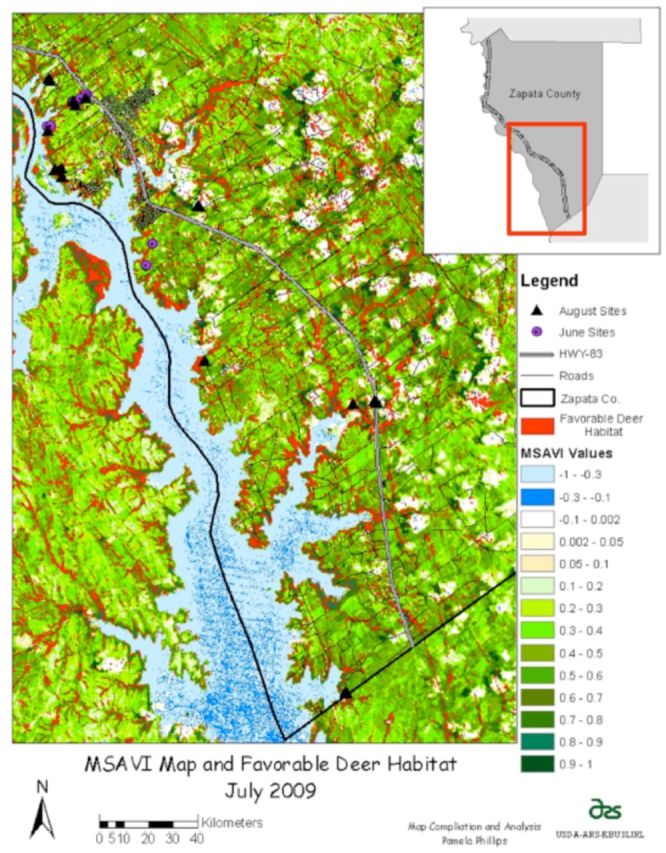
Landsat 5 Modified Soil Adjusted Vegetation Index (MSAVI) values for July 2009 with feature analyst analysis of favorable deer habitat for Zapata County, Texas. High quality figures are available online.

One hundred percent (10/10) of field sites designated,on 6 and 7 May 2008, by one of the USDA-APHIS-VS tick riders as being favorable deer habitat were a priori classified using the VLS Feature AnalystTM technique as favorable habitat ( Figure 5 , red areas) Open grass habitat was not classified as favorable deer habitat. Additionally, all sites classified a priori by satellite image analysis as favorable habitat for deer were positive for the presence of deer on 23–26 June and 10–12 August 2009. For areas in the study classified as favorable deer habitat, these typically had more than one factor for the presence of deer, i.e., bed sites, tracks, paths, and feces.

### Tick sampling

**Figure 6.**
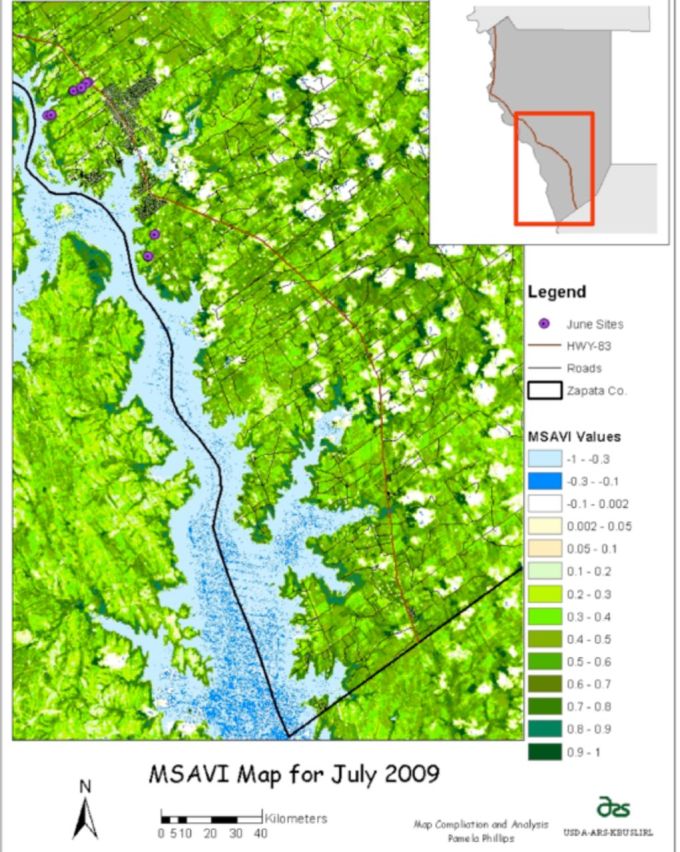
Modified Soil Adjusted Vegetation Index (MSAVI) for July with June sampling locations in Zapata County, Texas. High quality figures are available online.

**Figure 7.**
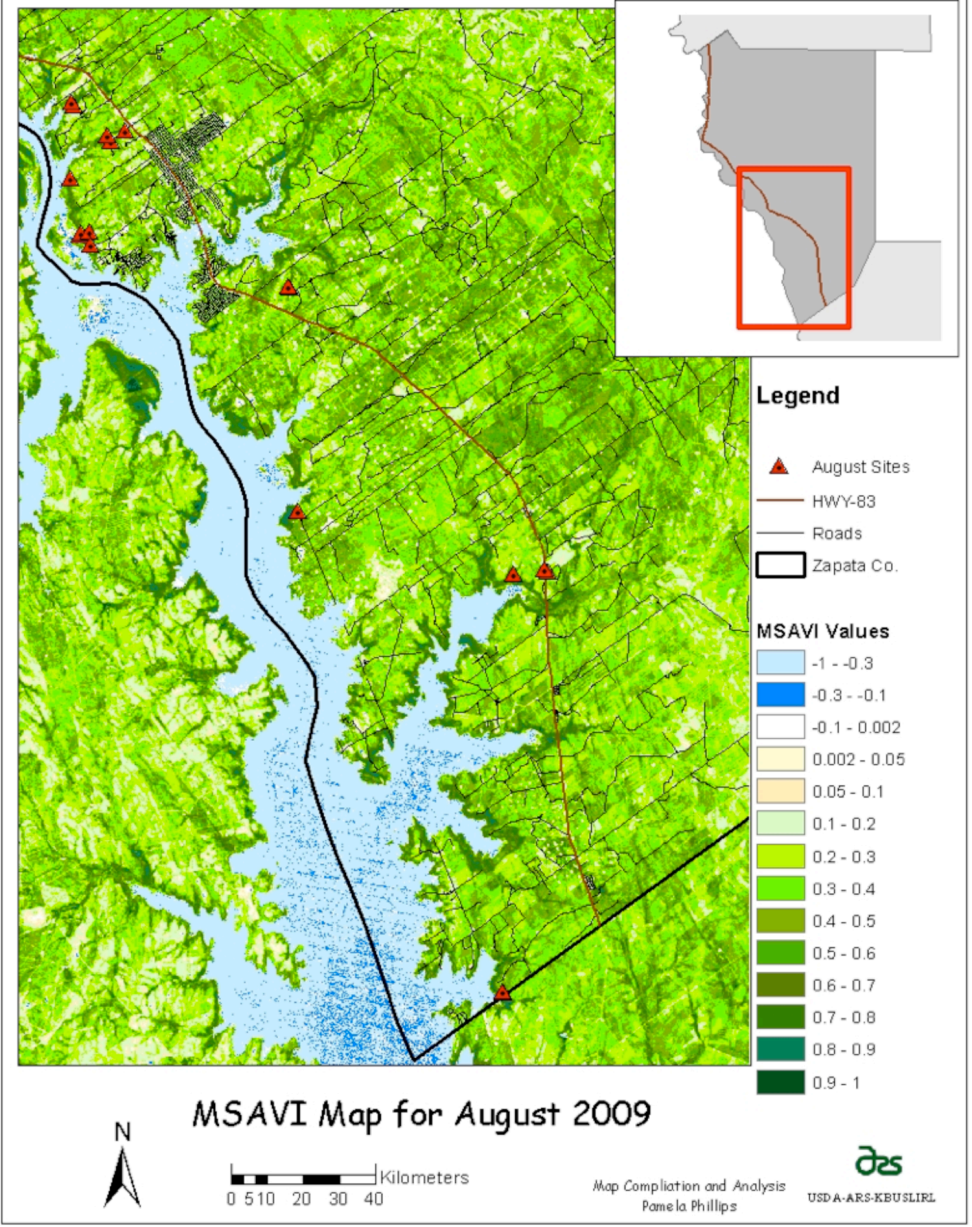
Modified Soil Adjusted Vegetation Index (MSAVI) for August with August sampling locations in Zapata County, Texas. High quality figures are available online.

In June, 713 *R. microplus* tick larvae were collected from 11 locations, and seven *Amblyomma* spp. were collected from one of the sample locations ( Table 2 , Figure 6 ). For June, the site with the highest number of tick larvae collected was brush habitat, and the site with the lowest number was open grasses. The August collection produced only 34 *R. microplus* larvae from 21 locations and seven *Amblyomma* spp. from three different locations ( Table 2 , Figure 7 ). The only locations producing ticks in August were dense or brush habitats ( Table 2 ), but it was not consistent throughout the sampling sites. *R. microplus* comprised 98% of the ticks sampled and *Amblyomma* spp. 2%.

### Weather data

The accumulated rainfall 30 days prior to sampling in June was 63.5 mm and only 0.3mm 30 days prior to sampling for larvae in August. August 2009 was very dry, with high temperatures over 38°C (100°F) each day in the field.

### Statistical model

The independent variables in the model predicting log tick counts were habitat, the MSAVI index (from July), distance from vegetation cover (decreasing log tick count with increasing distance), and a dummy variable that increased the three Volpe Pasture site log tick counts ( Table 4 ). The over-dispersion parameter was estimated to be 3.4, indicating moderate over-dispersion. This parameter was interpreted as capturing the spatially clumped distribution of larvae. While the other variables’ interpretation is straightforward, the negative slope for MSAVI requires comment ( Table 4 ). Note that the qualitative habitat variable adjusts the log mean of the tick count to the average for each habitat type. Thus, the MSAVI variable is a within habitat trend, meaning that within a given habitat, as the biomass increases, the log of the tick count decreases.

**Table 4.**
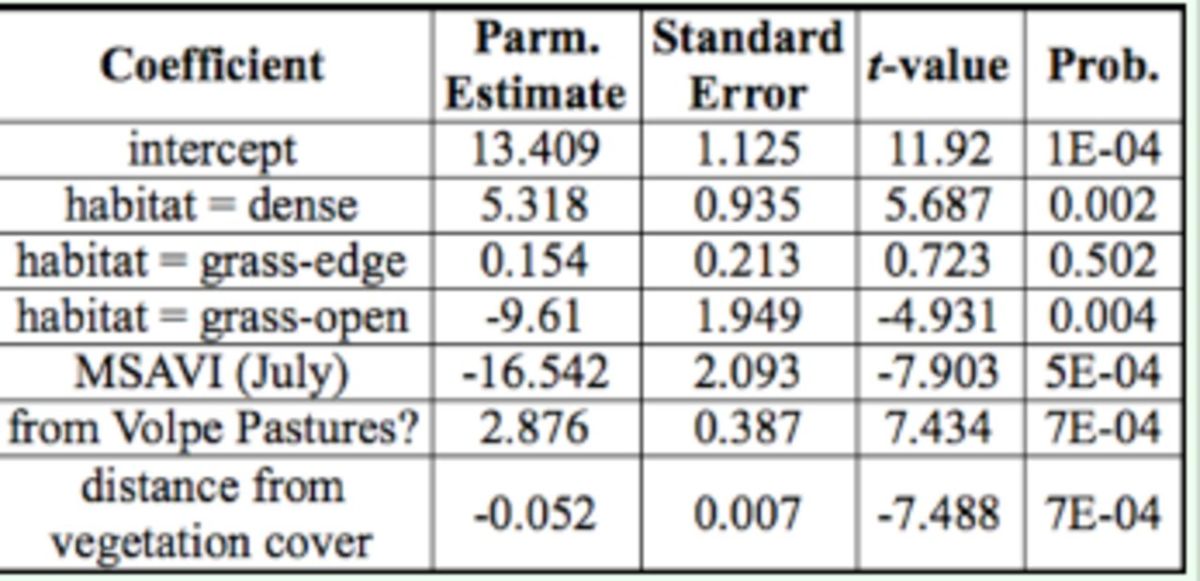
Coefficients of a generalized linear model with tick count data assumed to be samples from an over-dispersed Poisson distribution, with a log link (predictions are the logs of the count). Habitat variables are contrasts with habitat = brush

August data, with small (mostly zero) tick counts, indicated low larval tick survival (presence) in the field into August. Distance to water was not important for this area, perhaps because the Rio Grande River is the southern boundary for many of the properties sampled. The statistical model suggested that habitats where one is more likely to find deer are also places where ticks were more likely to occur.

While the model is simple, it demonstrates the importance of habitat (and, to some extent, microhabitat, e.g., deer trails) influences on tick abundance. Since habitat quality can be reliably estimated using satellite imagery data, this approach is useful for locating tick sampling sites (additional sites are needed to improve the statistical model and for model validation), ultimately to estimate tick abundances with reduced tick sampling. Efficient sampling to determine tick status requires knowing which locations are most likely to yield ticks if they are present.

## Discussion

Field sampling for *R. microplus* larvae was possible using a deer habitat classification model based on the analysis of Landsat 5 TM imagery. Higher larval counts were found in brush habitat and grasses that shared an edge with dense tree cover or dense brush. The lowest larval counts were in open grasses. Open grasses are typically clumped with patches of soil ( Figure 4 ), not providing much protection for the survival of tick larvae from high temperature, low humidity, and potential predators if they dropped off the host in these habitats. This is in agreement with Teel et al. (1996) , who characterized South Texas habitats of canopied mesquite and mixed-brush vegetation as more favorable for higher numbers and longer survival of cattle ticks than grass and grass-mixed brush. Davey et al. (1994) also found whether or not an area was favorable to *R. microplus* larvae survival was determined by temperature and humidity requirements, and these varied by season. In their study, they separated bufflegrass, *Cen-churus ciliarus* L., with no overstory (grass habitat) from bufflegrass with a mesquite, *Prosopis glandulosa* Torrey, overstory (shade habitat). In grass habitats, the survival was generally less than 15 days for seasons with low and high temperatures, and the larval survival in shaded habitats was < 25 days with low temperatures (Dec–Feb) and varied from 39 to 65 days for the other seasons (Mar– Nov). Teel et al. (2003) also found season to be the largest influence on detecting ticks in the field, followed by habitat. Vegetation type or habitat affects the microenvironment, making a site favorable or unfavorable for larval survival ( Fleetwood and Teel 1983 ; Sutherst et al. 1983 ; Teel 1984 ; Garris and Popham 1990 ). Although the macro weather data did not contribute to the model, as only June results were used, the dry conditions during the August sampling period appeared to decrease overall tick numbers. Effects of fluctuations in temperature and moisture levels on the biology and survival of *R. microplus* are well documented in the literature ( Tate 1941 ; Wilkinson 1953 ; Hitchcock 1955 ; Wilkinson and Wilson 1959 ; Cherny and de la Cruz 1971 ; Sutherst 1983 ; Sutherst et al. 1983 ; Utech et al. 1983 ; Sutherst and Maywald 1985 ).

Pound et al. (2010) commented there was a lack of field sampling for *Rhipicephalus (Boophilus)* tick species in South Texas, because once a tick is collected the property becomes an infestation and is quarantined. It is also recognized by the authors that transects without larval tick collections do not indicate the property is without an infestation. However, this analysis suggests that surveys of free-living ticks and how they relate to their host habitat will provide useful insights into the ecology of *Rhipicephalus (Boophilus)* ticks in South Texas. For *I. scapularis* , Goddard (1992) found that ticks cluster in areas related to deer trails or where deer forage. In this study, vegetation within or close to favorable deer habitat were more likely to harbor off-host *R. microplus* larvae regardless of vegetation type. The Volpe Pasture site sampled on 10 June had been vacated of cattle for several years and was only positive for the presence of deer at the time of our study. Open grasses had less favorable conditions for larval survival. Pound et al. (2010) also suggested the area of favorable habitat for white-tailed deer has increased, thus producing an increase in deer population, which has influenced the number of *R. microplus* tick infestations in South Texas. Historical analysis of satellite remote sensing data could provide background information on the changes in habitats and how they relate to tick eradication efforts.

In these analyses, NDVI and MSAVI equations were useful in distinguishing between areas of forage (brush) and grasses. MSAVI was slightly better at distinguishing these vegetation types. VLS Feature Analyst ^TM^ separated dense vegetation and vegetation along rivers better than either of the indices. Identifying habitat where ticks and their alternate host, white-tailed deer, are present may play a critical role in redeveloping successful efforts for future control of *R. microplus* .

As a result of our study, continued ecological studies of *R. microplus* ticks and their interaction with their hosts and the environment remain underway in Zapata County, Texas. How favorable deer and tick habitats change over the course of a year could provide insight into the epidemiology and seasonal activities of these ticks in South Texas. Permanent field sites have been established based on analysis of favorable deer and tick habitats. Each location has a Hobo ^®^ (Onset Computers, www.onsetcomp.com ) temperature and humidity data logger to monitor how these values vary within habitat and season. These sites are being sampled for larval ticks each month using the methodology described in this study. In addition, monthly MSAVI values will be processed and correlated to the temperature and humidity data. This will provide confidence in the comparison and correlation of concurrent seasonal patterns of larval tick numbers and satellite-derived vegetation indices.

Although our data set and sampling period were limited, it was clear from the analysis that larval ticks were most abundant in areas that harbor white-tailed deer. This study provided a technique for identifying locations where free-living larvae of cattle ticks are abundant based on preferred deer habitat identified using spatial analysis of satellite remote sensing. This approach should be useful for directing sampling activities in the field, ultimately enhancing future control strategies for cattle fever ticks.

## Abbreviations

CFTEP: Cattle Fever Tick Eradication Program
MSAVI: Modified Soil Adjusted Vegetation Index
NDVI: Normalized Difference Vegetation Index
